# Integrating Gaze Tracking and Head-Motion Prediction for Mobile Device Authentication: A Proof of Concept

**DOI:** 10.3390/s18092894

**Published:** 2018-08-31

**Authors:** Zhuo Ma, Xinglong Wang, Ruijie Ma, Zhuzhu Wang, Jianfeng Ma

**Affiliations:** 1 School of Cyber Engineering, Xidian University, Xi’an 710071, China; JMaris4733865@gmail.com (R.M.); jfma@mail.xidian.edu.cn (J.M.); 2 Shaanxi Key Laboratory of Network and System Security, Xidian University, Xi’an 710071, China; 3 ZTE Corporation, Xi’an 710114, China; wang.zhuzhu@zte.com.cn

**Keywords:** smart mobile devices, gaze tracking, head motions, authentication, neural networks

## Abstract

We introduce a two-stream model to use reflexive eye movements for smart mobile device authentication. Our model is based on two pre-trained neural networks, *iTracker* and *PredNet*, targeting two independent tasks: (i) gaze tracking and (ii) future frame prediction. We design a procedure to randomly generate the visual stimulus on the screen of mobile device, and the frontal camera will simultaneously capture head motions of the user as one watches it. Then, *iTracker* calculates the gaze-coordinates error which is treated as a *static feature*. To solve the imprecise gaze-coordinates caused by the low resolution of the frontal camera, we further take advantage of *PredNet* to extract the *dynamic features* between consecutive frames. In order to resist traditional attacks (shoulder surfing and impersonation attacks) during the procedure of mobile device authentication, we innovatively combine *static features* and *dynamic features* to train a 2-class support vector machine (SVM) classifier. The experiment results show that the classifier achieves accuracy of 98.6% to authenticate the user identity of mobile devices.

## 1. Introduction

In the era of the Mobile Internet, a large amount of private information is stored in smart mobile devices [1,2,3,4,5,6], which make the authentication of users a vital precondition of the secure access to the sensitive data. A traditional password authentication method has some negative characteristics such as shoulder-surfing [7]. In later studies, several biometric techniques have been applied to user authentication. For example, Sin et al. proposed fingerprint recognition systems based on template updating [8]; Lin et al. utilized the robust thin plate spline (RTPS) to achieve the user identification [9]; Parkhi et al. [10] and Ning et al. [11] developed face recognition methods using Convolutional Neural Network (CNN) architectures and biomimetic uncorrelated locality discriminant projection (BULDP), respectively. Although fingerprint and face recognition methods can defend shoulder-surfing, impersonation attacks still exist [12].

Recently, eye tracking has been used in some emerging fields such as human–computer interaction [13,14,15,16] and computer vision [17,18] as an important technique across many domains with a series of decent research results. Among those results, an authentication method exploiting gaze-based information is easy to implement relying on the high precision of dedicated devices. Meanwhile, some researchers [19] used stereoscopic views with multiple devices or light sources to achieve eye tracking, whereas, it is difficult to apply these equipment-based eye tracking methods to mobile devices.

**Figure 1:**
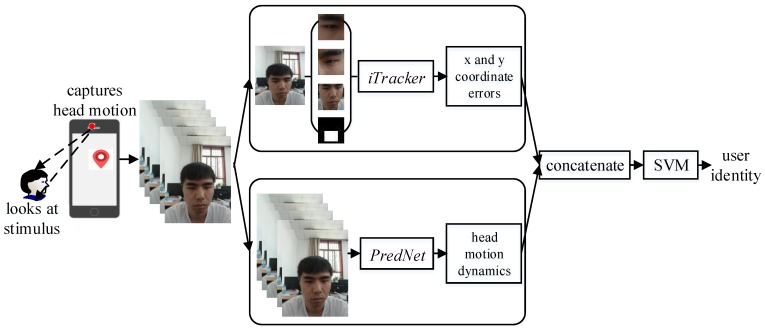
Authentication workflow.

In view of the above questions, this paper proposes a novel mobile device authentication method which integrates the gaze tracking and head-moving prediction. The whole procedure of our method is shown in Figure 1. First, the smart mobile device randomly shows an interactive visual stimulus on the screen, which is referred to as *gaze–challenge*. Simultaneously, the front camera captures head motions of the user as he/she watches the screen. Then, this system adopts two kinds of deep neural networks to extract gaze features that can be divided into two categories, one using a convolutional neural network to extract *static features* that rely on patterns where the user is looking, and another using a recurrent convolutional neural network to extract *dynamic features* that depend on patterns of how the user’s head is moving. Finally, the smart mobile device combines *static features* and *dynamic features* that are used to train a dedicated two-class Support Vector Machine (SVM) classifier and make a decision about the user’s identity which is referred to as *gaze–response*.

In the process of feature extraction, we consider using the *iTracker* [20] to calculate the error between the prediction and the ground truth of gaze coordinates cast on the screen. It can provide a tiny circle whose radius is the value of error, and which is surrounded around the visual stimulus to determine the movement of gaze. Obviously, the random stimulus trajectory ensures that the gaze maintains freshness to avoid the impersonation attack. In other words, if there are some predicted coordinates beyond the tiny area, the gaze might be invalid or even fake. Therefore, we refer to the two errors of abscissa and ordinate, respectively, as *static features*.

*Static features* give a fresh gaze trajectory but is weak at providing the information of identification. To enhance the features of identification when the user’s head moves along with the visual stimulus, we adapt *PredNet* [21] to extract dynamic representation that is used to determine the user’s identity. *PredNet* [21] applies the existing video frame sequence to predicting the future frame. The *Representation* module extracts the *dynamic features* from consecutive frames, so we utilize *dynamic features* to identify valid users. In conclusion, integrating *static features* and *dynamic features* not only defends the impersonation attacks but also provides an efficient identity recognition.

## 2. Related Work

Human visual system is reflexive and fast, and different people possess different gaze information, which has attracted great attention in the biometric authentication field. Over the past two decades, plenty of gaze tracking methods have emerged and been used in the medical field, but their utilization in attack detection and authentication field has rarely been found until recent years. Two attack scenarios were considered by Komogortsev et al. [22] of whether the imposter had access to the biometric database. Their results suggested that eye movement biometrics were highly resistant to circumvention by artificial recordings. A novel method for face liveness detection by tracking the gaze of the user with an ordinary webcam was proposed by Ali et al. [23] to resist spoofing attacks on biometric systems. A summary of works on the authentication methods and systems previous to 2010 were given by Zhang et al. [24], while the methods and results of gaze tracking authentication systems provided in works of recent years were compared carefully in Saeed [25]. Zhang et al. [26,27,28] presented a person-independent eye gaze interface that immediately supported spontaneous interaction with displays, without any prior user calibration or training. In their work, localisation of inner eye corners and eye centres was used to realize calibration-free interaction and gaze tracking. Moreover, the existing works can be classified into two types, of which one takes gaze tracking as a human–computer interaction interface and utilizes gaze patterns as Personal Identification Numbers (PINs) to access authentication systems, and the other exploits the classification results concluded by universal gaze features that are extracted from several gaze patterns of individual people to differentiate one user from another.

The first type of authentication method needs users to stare at the screen so that the secret information can be covertly inputted in a natural way, where the secret information is usually in the form of passwords [29,30,31] or other distinct ways [32,33,34]. The work of Chen et al. [35] belongs to the first method, taking gaze tracking as security primitives. It endows authentication systems with some additional advantages including but not limited to the protection from shoulder-surfing and smudge attacks. However, this authentication method is still in need of some memorization of secret information and as a result suffers from some latent attacks such as reply attacks.

The work of Sluganovic et al. [36] belongs to the second method, making use of the different biometric gaze features of different people to distinguish different users. An authentication system equipped with this method is more friendly to use since the users no need to remember any secret information, and can also defend impersonation attacks due to the unique gaze characteristics. Nowadays, the existing works using this method always have a high cost because of the use of expensive invasive gaze tracking devices. Consequently, we aim to make the gaze tracking authentication approach a more pervasive and available technology by only using the front camera in smart phones rather than high cost gaze tracking devices. In the rest of this paper, we will introduce our authentication procedure in detail.

## 3. Authentication Procedure

The authentication approach is summarized in Figure 1 inspired by [37,38,39,40]. We can divide the work into five subsections: (i) generating interactive visual stimulus; (ii) preprocessing head-moving frames; (iii) extracting *static features*; (iv) extracting *dynamic features* and (v) classifying user’s identity. The individual pieces are described in turn in the following subsections.

### 3.1. Generating Interactive Visual Stimulus

The smart mobile phone shows a randomly interactive visual stimulus on the screen. The stimulus’ motions should conform to the moving habits of human eyes. Therefore, we start with a short background of the human visual system. Even when one’s gaze is firmly fixated on a single stimulus, human eyes are never completely still. They are constantly making hundreds of micro movements per second, which are interlaced with more than 100,000 larger movements during the course of one day [41]. During visual tasks, such as search or scene perception, our eyes alternate between *fixations* and *saccades*. *Fixations* are used to maintain the visual focus on a single stimulus, while *saccades* reorient the eye to focus the gaze on the next desired position [36].

**Figure 2:**
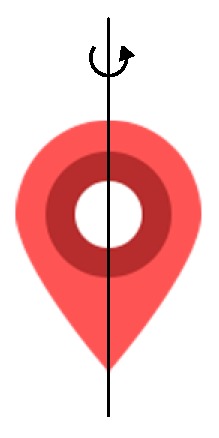
Visual stimulus icon.

Inspired by the above description, we design a visual stimulus icon that can rotate around the vertical central axis in Figure 2. Instead of showing a still icon, we show a rotated icon when it stays at a fixed position on the screen, which directs the fixation of the gaze to lie in the middle of the icon. The white central circle of icon is different from the red surroundings, which can also attract the user’s attention on the center point of the icon.

**Figure 3:**
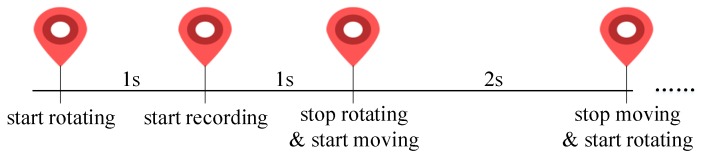
Temporal movements of the stimulus.

**Figure A1:**
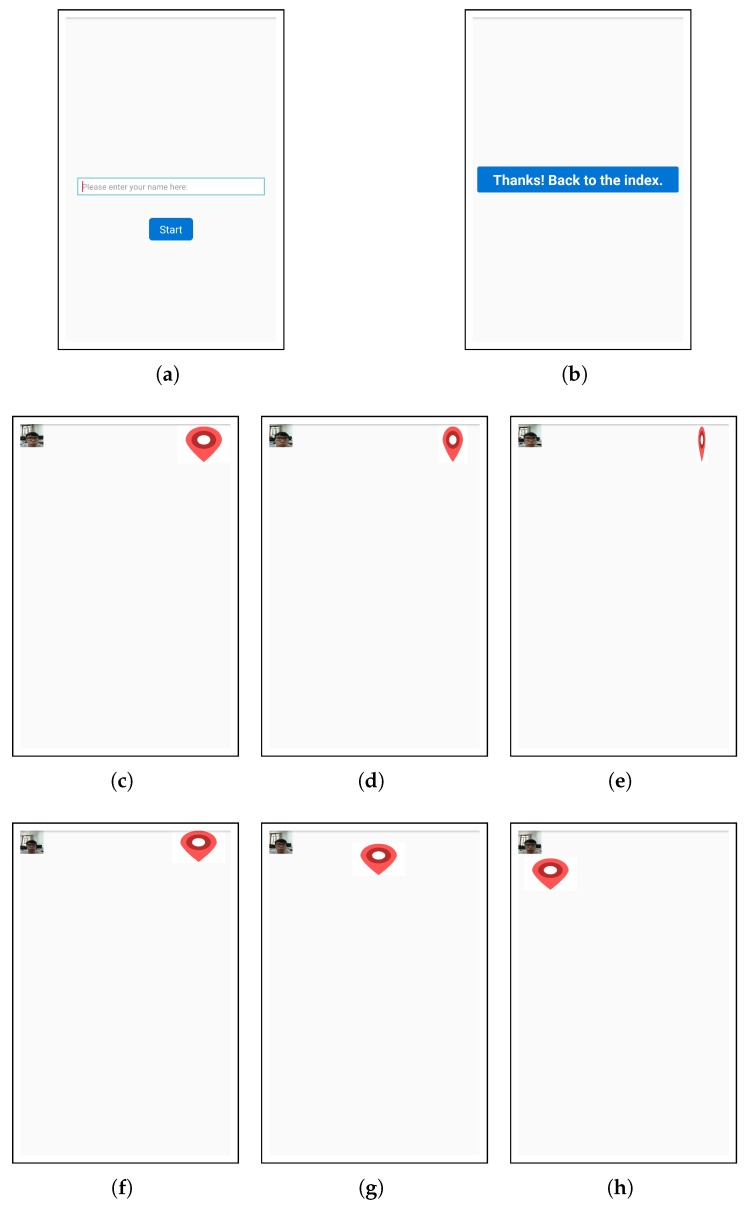
Portions of Android layouts. (**a**) instructs the user to input his/her name and start paying attention and fixating on the stimulus icon. (**b**) shows the end of the procedure. (**c**–**e**) shows the rotation procedure at three discrete time points. (**f**–**h**) shows the movement procedure in three discrete time points. Moreover, in order to help the user ensure that his/her face can be captured by the front camera, we put a *SurfaceTexture* component in the top left corner of the screen.

The stimulus movements are represented in Figure 3. First of all, to avoid distraction from notifications, we ensure that the user applies *Airplane Mode* with no network connection throughout the task, until the task is complete. The icon rotates at a random position in the beginning and then moves to the next position with a random orientation, each of the motions takes 2 s and alternates. We start the recording after 1 s; in this way, the mussy gaze scanning in the first second can be wiped off. Moreover, the recording frequency is 25 fps. Last but not least, the user needs to ensure that his/her face is visible in the front camera. This is critical as we do not hope to track where someone is looking without a picture of the face. For portions of Android layouts, please refer to Figure A1 in Appendix A in detail.

**Figure 4:**
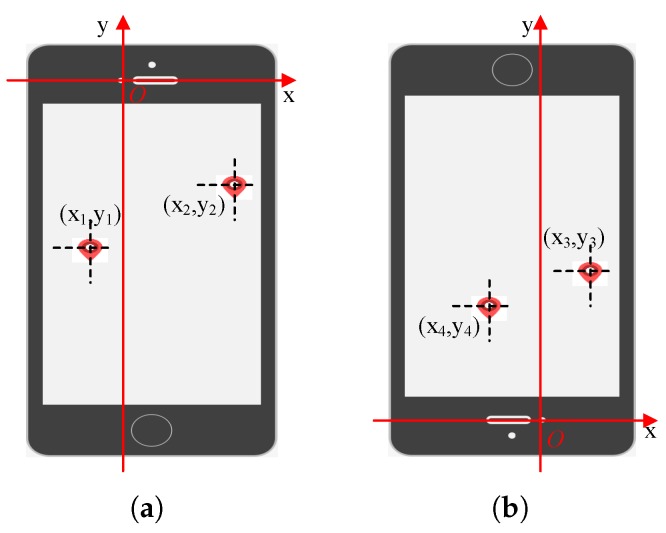
The coordinate axis takes the front camera as the ordinate origin. (**a**) Normal orientation of smart phone; (**b**) Orientation of smart phone is inverted.

When eyes fixate on the rotated stimulus at a fixed position on the screen, we predict the gaze coordinates relative to the front camera taken as the ordinate origin that are shown in Figure 4. In Figure 4a, $\left(x_{1},y_{1}\right)$ is located in the third quadrant and $\left(x_{2},y_{2}\right)$ is located in the second quadrant, so $x_{1},y_{1},y_{2}<0$ and $x_{2}>0$. An inverted screen is represented in Figure 4b, $\left(x_{3},y_{3}\right)$ is located in the first quadrant and $\left(x_{4},y_{4}\right)$ is located in the fourth quadrant, so $x_{3},y_{3},y_{4}>0$ and $x_{4}<0$. It is also beneficial to keep data variability in the pictures when users change the orientation of their mobile devices to be inverted.

### 3.2. Preprocessing Head-Moving Frames

**Figure 5:**
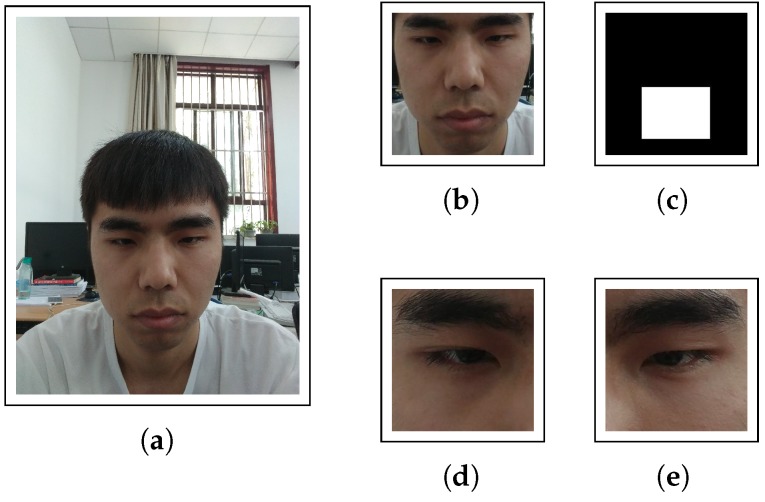
Image data after preprocessing to satisfy the input demand of the neural network in the next subsection; (**a**) original image; (**b**) face; (**c**) face grid; (**d**) right eye; (**e**) left eye.

During the preprocessing duration, we crop the image of eyes and faces to satisfy the input demand of the neural network in Section 3.3 by adopting the interface provided by OpenCV. After preprocessing, we can obtain the following images shown in Figure 5. We need to emphasize face grid that is a binary mask used to indicate the location and size of the head within the original image while the face, left eye and right eye are simply detected and cropped from the original image.

### 3.3. Extracting Static Features

**Figure 6:**
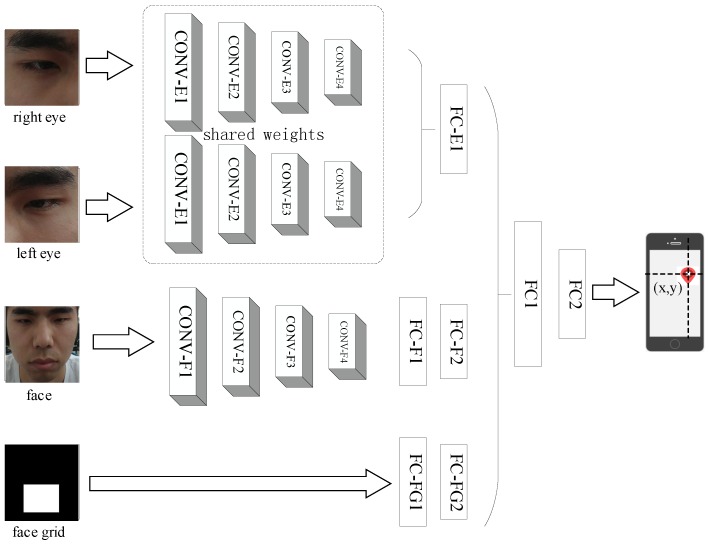
Convolutional neural network architecture named *iTracker* [20].

*iTacker* [20] is an end-to-end CNN for robust eye tracking shown in Figure 6. Inputs include right eye, left eye and face images of size 224×224 and face grid of size 25×25. In addition, the distance between the user and the smart mobile device can be measured and calculated by *iTracker* because the area of the face grid will decrease when the subject is far away from the screen. Otherwise, the area will increase. The relationship between them is shown in Table 1. It can be seen that there is a linear relationship between the distance from head to screen and the area of the face grid. Therefore, we can infer the position of the head based on the size of the face grid. On this basis, we can perform a calibration of distance using the face grid information. In addition, it can enhance the variety of the dataset when the subject observes the stimulus from different distances. Thus, we adopt the various distance samples to *iTracker* to fine-tune a relatively advantageous calibration effect.

Parameters of each convolutional layer are shown in the Table 2 and the number of neurons of each full-connected layer are shown in Table 3. The original output is the Euclidean distance, in centimeters, from the front camera. However, we adapt the output to distances between the predicted gaze coordinate and the ground truth along the *x*-axis and *y*-axis respectively, which act as *static features*.

### 3.4. Extracting Dynamic Features

**Figure 7:**
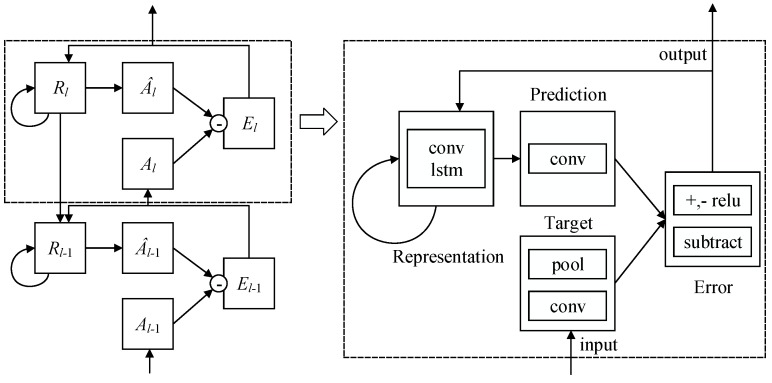
Recurrent convolutional neural network architecture named *PredNet* [21].

*PredNet* [21] can predict the future frames in a video sequence by learning about the structure of the visual world shown in Figure 7. We review the relations between each modules of *PredNet* [21] in the following formulas ($x_{t}$ denotes a sequence of images, *t* denotes the time, and *l* denotes the layer):

(1) $$\begin{array}{cc} A_{l}^{t} & =\left\{\begin{array}{ccc} x_{t}, & \mathrm{if} & l=0,\\ MaxPool\left(ReLU\left(Conv\left(E_{l-1}^{t}\right)\right)\right), & \mathrm{if} & l>0, \end{array}\right. \end{array}$$

(2) $$\begin{array}{cc} {\hat{A}}_{l}^{t} & =\left\{\begin{array}{ccc} SatLU\left(ReLU\left(Conv\left(R_{l}^{t}\right)\right)\right), & \mathrm{if} & l=0,\\ ReLU\left(Conv\left(R_{l}^{t}\right)\right), & \mathrm{if} & l>0, \end{array}\right. \end{array}$$

(3) $$\begin{array}{cc} E_{l}^{t} & =\left[ReLU\left(A_{l}^{t}-{\hat{A}}_{l}^{t}\right);ReLU\left({\hat{A}}_{l}^{t}-A_{l}^{t}\right)\right], \end{array}$$

(4) $$\begin{array}{cc} R_{l}^{t} & =ConvLSTM\left(E_{l}^{t-1},R_{l}^{t-1},UpSample\left(R_{l+1}^{t}\right)\right). \end{array}$$

**Figure 8:**
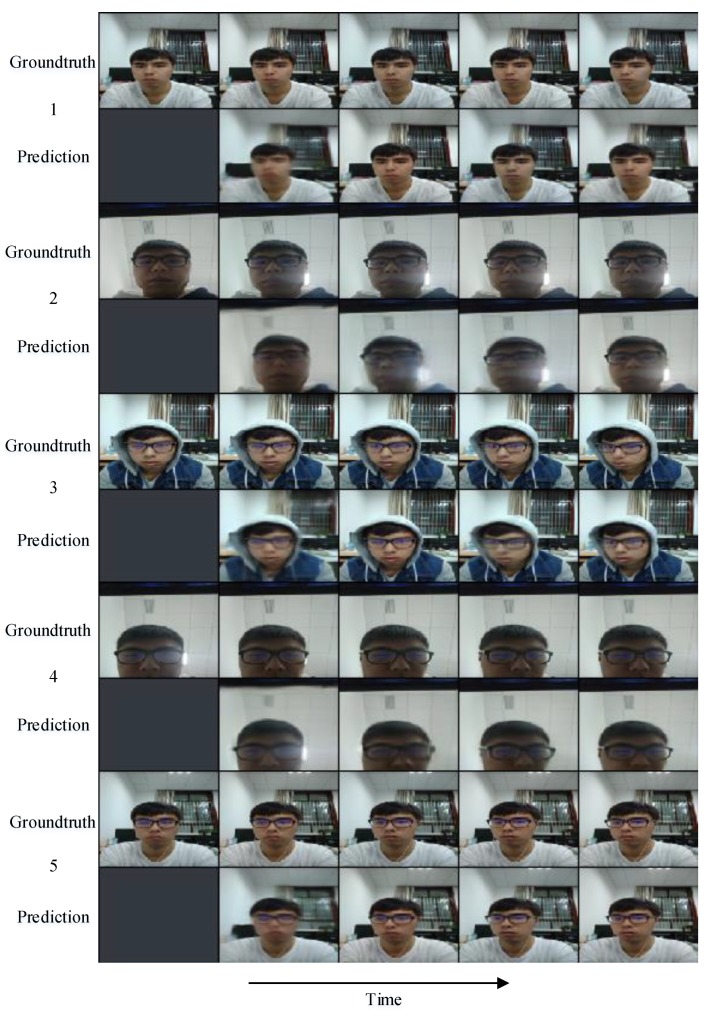
Comparisons between ground truth and prediction.

**Figure 9:**
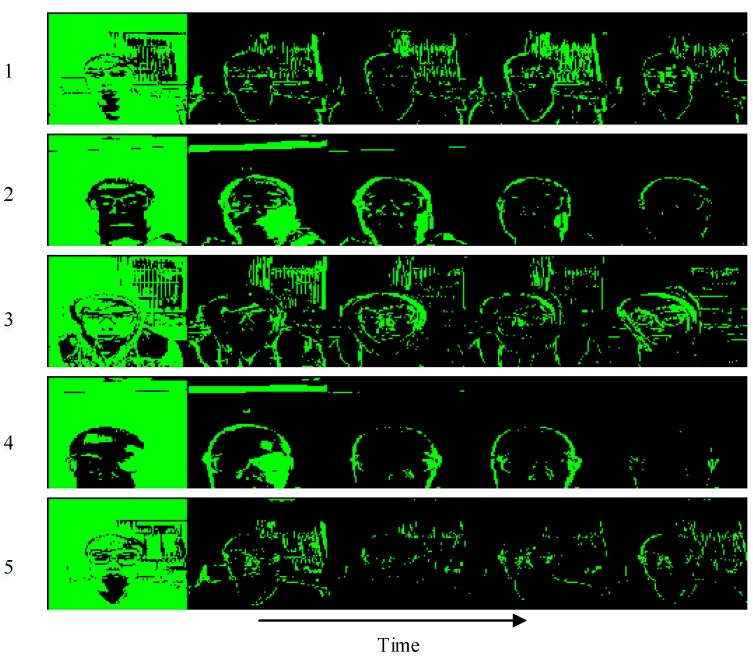
Difference between ground truth and prediction. The green color denotes that the absolute value of subtraction is greater than the threshold and the black region corresponds to the absolute value of subtraction being less than the threshold. The threshold is set as 0.1.

**Figure 10:**
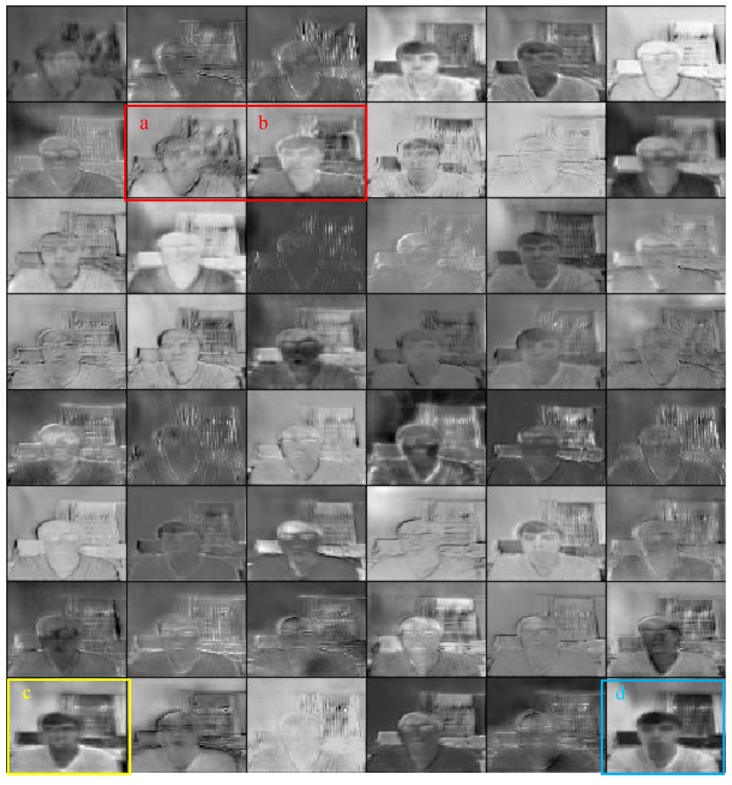
Visualization of the different channels of a representation module.

We adopt a 4-layer *PredNet* [21] model to the consecutive head-moving frames and illustrate five comparisons in Figure 8 and then calculate the difference between ground truth and prediction shown in Figure 9. It can be inferred from Figure 9 that the prediction is more approximate to the ground truth as time goes by. As for the representation module in *PredNet* [21], whose tensor field was proved to generalize well to other classification tasks, we can utilize this dynamics tensor coding as *dynamic features* to identify the valid user and complete the authentication task. Our representation module of *PredNet* learns to predict future frames in *dynamic features* of a video sequence. Each layer in the module makes local predictions and only forwards the differences between predictions and ground truth from those predictions to subsequent network layers. The module learns internal representations that are useful for decoding latent object parameters (e.g., head motion) that support object recognition with fewer training views. To possess more knowledge of the representation module, we refer to [42,43,44,45,46] to learn that the representation module acts as an upsampling method to reconstruct the scale of feature maps and identifies with a deconvolutional network to revivify the original images. Therefore, we visualize a second layer of a representation module of Group 1 data in Figure 10, we can see: the head turns right a little bit from the a channel to b channel, the squint of head motion appears in the c channel, and an approximate intensity simulation exists in the d channel.

### 3.5. Classifying User’s Identity

**Figure A2:**
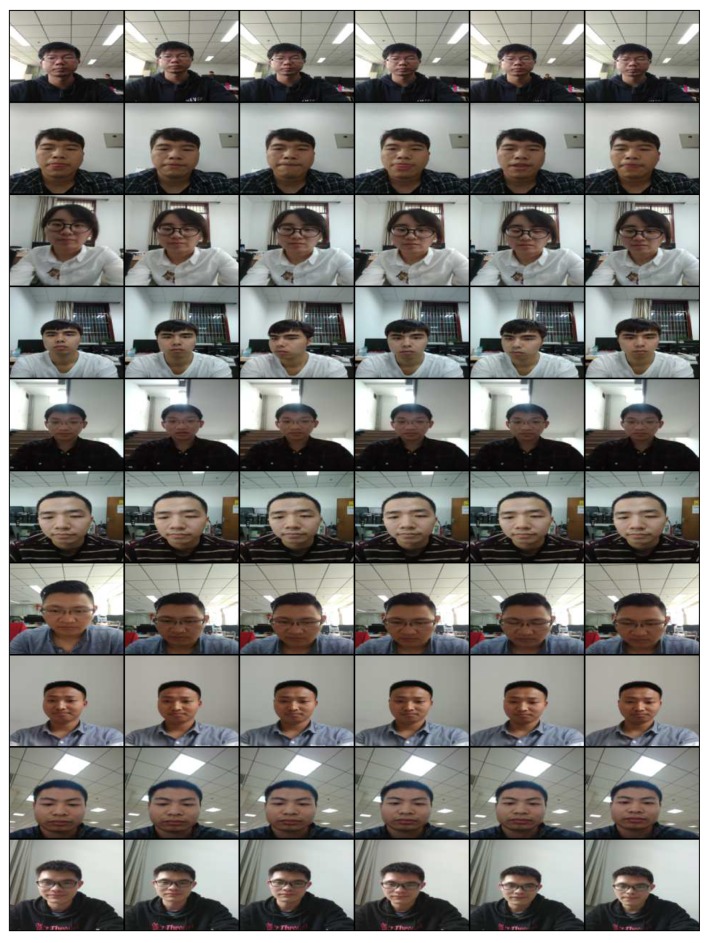
Sample frames from the whole dataset.

*Static features* and *dynamic features* are concatenated and then labelled in line with the user’s identity. Finally, features and labels are fed into an SVM [47] algorithm to train a dedicated classifier to identify the valid user. For head-moving samples, please refer to Figure A2 in Appendix A. We adopt the interface provided by scikit-learn [48,49] to model a two-class classifier by cross-validation and fit the test dataset. It is critical for the valid user to participate more in data collection to solve the data-imbalanced problem. The detailed experiment results are shown in Section 4.

## 4. Results

There are 37 distinct participants (26 males and 11 females, 14 teachers and 23 students) aged between 22–38 years (mean = 26.5, std = 3.4) that are involved in our experiments. Among them, 36 participants belong to invalid users and one participant is the valid user because each phone device generally has one owner. The facial videos are recorded with 25 fps using the mobile phone. Each invalid user repeats the experiment four times and each experiment produces a sequence of 500 frames. That is, the length of the collection time for each sequence lasts 20 s. In order to solve the imbalance problem of the dataset, the valid user repeats the experiment 144 times. Therefore, the dataset consists of 288 groups of the frame sequence. A sequence is labelled 1.0 if it belongs to the valid user; otherwise, it is labelled −1.0.

In order to ensure the reliability of the experimental results, we adopted the three-fold cross-validation. Specifically, we first randomly split the whole dataset into four equally sized segments, i.e., the training set is 216 and the test set is 72. Three segments are used to train a model and the rest is employed to test it. In the procedure of cross-validation, the training and testing datasets must be crossed over in successive rounds such that each frame sequence trial has a chance of being invalidated. It is reasonable to adopt a grid search to find the best hyper-parameters of SVM [47] shown in Table 4. It takes 3.5 s to finish feature processing, and, in some significant authentication systems, which care more about attacks by imposters such as shoulder surfing and impersonation attacks, the time consumption is absolutely tolerable.

Our experiments show that hyper-parameters of the best classifier are kernel = ‘linear’ and C=0.001. Therefore, we apply this classifier to the test set and obtain the following confusion matrix shown in Table 5.

**Figure 11:**
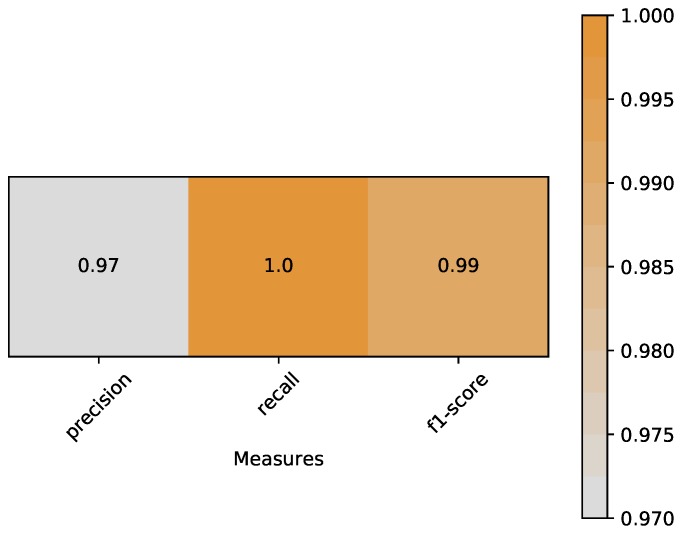
Heat map of measures include *precision*, *recall* and *f1-score*.

**Figure 12:**
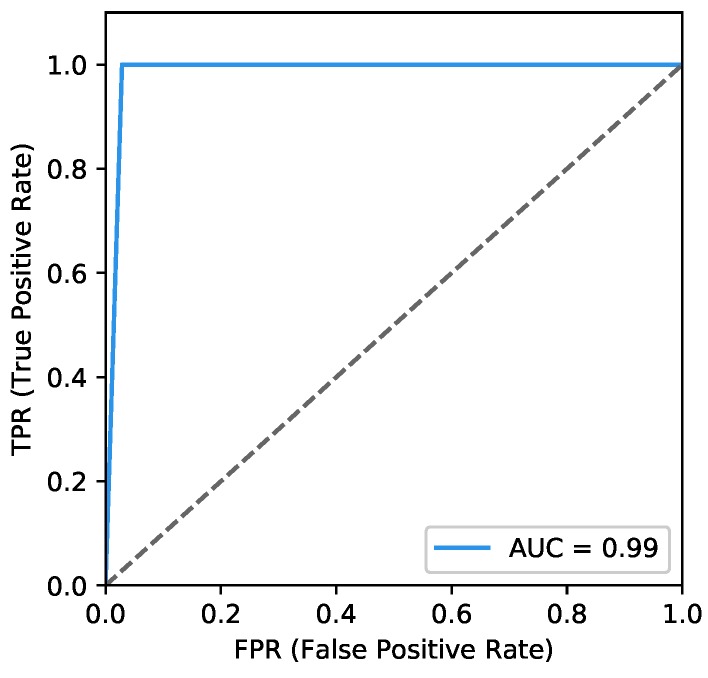
*ROC* curve.

According to the confusion matrix, we can infer that the *TP* (true positive) is 37, *FP* (false positive) is 1, *FN* (false negative) is 0 and *TN* (true negative) is 34. Finally, we calculate the *accuracy*, *precision*, *recall*, *f1-score*, *AUC* and plot the *ROC* curve to report the classification performance:

1. *Accuracy* is the most intuitive performance measure and it is simply a ratio of correctly predicted observation to the total observations. Based on Equation (5), *accuracy* of our classifier is 0.986:
  (5) $$Accuracy=\frac{TP+TN}{TP+TN+FP+FN}.$$
2. *Precision* is the ratio of correctly predicted positive observations to the total predicted positive observations. Based on Equation (6), *precision* of our classifier is 0.97:
  (6) $$Precision=\frac{TP}{TP+FP}.$$
  *Recall* is the ratio of correctly predicted positive observations to the all observations in true class. Based on Equation (7), *recall* of our classifier is 1.0:
  (7) $$Recall=\frac{TP}{TP+FN}.$$
  *F1-score* is the weighted average of Precision and Recall. Therefore, this score takes both false positives and false negatives into account. Based on Equations (8) and (9), *f1-score* of our classifier is 0.99:
  (8) $$\begin{array}{c} \frac{2}{F1}=\frac{1}{P}+\frac{1}{R}, \end{array}$$
  (9) $$\begin{array}{cc} \Rightarrow & F1=\frac{2TP}{2TP+FP+FN}. \end{array}$$
  A heat map is plotted in Figure 11 to represent the above measures clearly.
3. *ROC* curves typically feature a true positive rate (Equation (10)) on the *y*-axis, and the false positive rate (Equation (11)) on the *x*-axis. This means that the top left corner of the plot is the ideal point where a false positive rate of zero and a true positive rate of one. It does mean that a larger area under the curve (*AUC*) is usually better. We plot the *ROC* curve of our classifier in Figure 12. The area of *AUC* is labelled in the bottom right corner:
  (10) $$\begin{array}{cc} TPR & =\frac{TP}{TP+FN}, \end{array}$$
  (11) $$\begin{array}{cc} FPR & =\frac{FP}{FP+TN}. \end{array}$$

**Figure 13:**
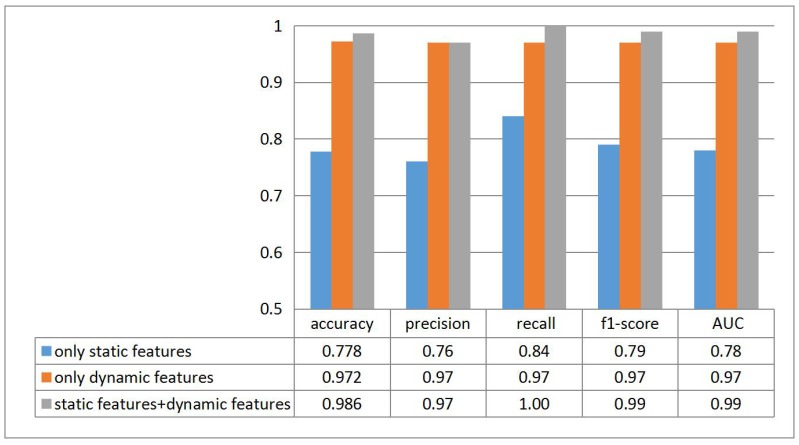
Contributions of different types of features.

To observe the contribution of different features, we experiment on both single and combined features, the results are shown in Figure 13. It is discriminative that *dynamic features* are more helpful than *static features* and the combined features cover the benefits of both.

**Figure 14:**
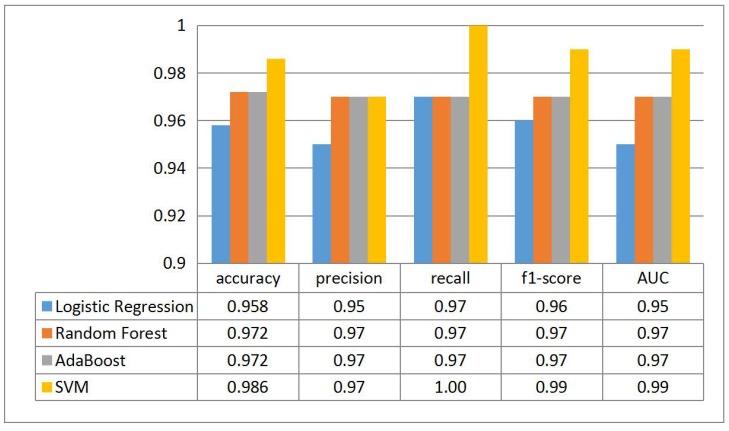
The effects of different classification algorithms.

To validate that the SVM [47] algorithm we choose in the work is ideal, we also check other classification algorithms and represent the results in Figure 14. It is obvious that SVM possesses the optimal performance while the Random Forest and AdaBoost provide the same statistics on the test set.

Finally, we compare our method with other pervasive authentication methods in Table 6. Of course, it is injudicious to use False Positive Rate (FPR) and False Negative Rate (FNR) to compare our method with a password and increasingly accurate fingerprints and face recognition. As we all know, we can enter the authentication system effortlessly if we remember the login password, so both FPR and FNR of passwords are 0.00. In particular, we adopt our dataset to state-of-the-art fingerprints [8] and the face recognition [10] algorithm. It is noticeable that our method is able to resist shoulder surfing and impersonation attacks, and achieve an acceptable result in terms of accuracy at the same time.

We have a simple test that image and video data of 16 students are trained respectively by the deep face recognition [10] and our authentication method. As can be seen in Table 7, 16 videos of the corresponding participants recorded in advance are used to find out whether the impersonation can be detected by these two methods. The error rate of 68.75% indicates that general face recognition methods have difficulty dealing with impersonation attacks. The random gaze–challenge and the corresponding gaze–response of our method guarantee authentic human behaviors. Thus, the videos recorded in advance can be detected. As a result, the novel authentication method we proposed combines gaze tracking and head-moving prediction to determine user identity. We provide higher security since the traditional biometric authentication methods are easily cheated by impersonation attacks.

## 5. Conclusions

In this paper, we proposed a novel method to use reflexive eye movements for smart mobile device authentication. Inspired by a two-stream neural network that has become a pervasive domain in recent years, we utilize *iTracker* [20] to extract the location of gaze and *PredNet* [21] to extract the dynamics of head motions when users are tracking the randomly interactive visual stimulus. Due to the fact that human eyes are fast, reflexive, responsive, and carry information unique to other individuals, attempting to apply gaze patterns to authentication is particularly attractive. Moreover, dynamics of head motions facilitate the authentication system to become more robust.

In the experiment, 288 groups of frame sequence data were collected from all the students and teachers in our lab. Indeed, we know that the insufficient sample data were not conducive to the accuracy of the model, but the main contributions of this paper are to guarantee the authentic human behaviors to resist impersonation attacks, and elucidate a proof of concept prototype implementing the fundamental concepts of a new authentication method by integrating gaze tracking and head-motion prediction. Using *static features* and *dynamic features* improves the recognition rate in spite of the small sample data set. The accuracy of our method is 98.6% by the complementary features provided by *iTracker* [20] and *PredNet* [21]. Furthermore, simplifying the input format and giving a deeper understanding of features extracted by the architecture are important future directions.

## Figures and Tables

**Table 1:** Relationship between distances from head to screen and the black area (area of the face grid is always 25×25 pixels).

Distance from head to screen (cm)	10	20	30	40
**Area of black**	≈16×16	≈12×12	≈8×8	≈4×4

**Table 2:** Parameters of each convolutional layer.

Name	Filter Size	Number of Kernel
CONV-E1	11 × 11	96
CONV-F1	11 × 11	96
CONV-E2	5 × 5	256
CONV-F2	5 × 5	256
CONV-E3	3 × 3	384
CONV-F3	3 × 3	384
CONV-E4	1 × 1	64
CONV-F4	1 × 1	64

**Table 3:** Number of neurons of each full-connected layer.

Name	Number of Neurons
FC-E1	128
FC-F1	128
FC-F2	64
FC-FG1	256
FC-FG2	128
FC1	128
FC2	2

**Table 4:** Hyper-parameters of SVM.

Hyper-Parameter	Explanation	List of Values
kernel	kernel function	[‘rbf’, ‘linear’]
*C*	cost parameter	[1 $\times 10^{-07}$, ..., 1, 10, 100]
gamma	hyper-parameter of RBF (only if kernel = ‘rbf’)	[1 $\times 10^{-05}$, ..., 1, 10, 100]

**Table 5:** Confusion matrix.

	Prediction	1.0	−1.0
ground truth			
**1.0**		37	0
**−1.0**		1	34

**Table 6:** Comparison between different authentication methods.

Authentication Method	Accuracy	FPR	FNR	Resist Shoulder Surfing?	Resist Impersonation Attacks?
Password	100.0%	0.00	0.00	No	No
Fingerprint [8]	98.6%	0.00	0.01	Yes	No
Face recognition [10]	100.0%	0.00	0.00	Yes	No
Our method	98.6%	0.01	0.00	Yes	Yes

**Table 7:** A test against the impersonation attack.

Authentication Method	Accepted Impersonation	Rejected Impersonation	Error Rate
Deep face recognition [10]	11	5	68.75%
Our method	0	16	0.00%

## Abbreviations

The following abbreviations are used in this manuscript:

SVM	Support Vector Machine
CNN	Convolutional Neural Network
PINs	Personal Identification Numbers
TP	True Positive
FP	False Positive
FN	False Negative
TN	True Negative
AUC	Area Under Curve
ROC	Receiver Operating Characteristic Curve
TPR	True Positive Rate
FPR	False Positive Rate
FNR	False Negative Rate

## Appendix A

Figure A1 represents portions of Android layouts.

Figure A2 shows a part of the consecutive head-moving sequence samples.

## References

[1] Wu D., Yan J., Wang H., Wu D., Wang R. (2017). Social Attribute aware Incentive Mechanisms for Video Distribution in Device-to-Device Communications. IEEE Trans. Multimedia.

[2] Wu D., Liu Q., Wang H., Wu D., Wang R. (2017). Socially Aware Energy Efficient Mobile Edge Collaboration for Video Distribution. IEEE Trans. Multimedia.

[3] Wu D., Si S., Wu S., Wang R. (2017). Dynamic Trust Relationships Aware Data Privacy Protection in Mobile Crowd-Sensing. IEEE Internet Things J..

[4] Jiang Q., Chen Z., Li B., Shen J., Yang L., Ma J. (2017). Security analysis and improvement of bio-hashing based three-factor authentication scheme for telecare medical information systems. J. Am. Intell. Humaniz. Comput..

[5] Jiang Q., Zeadally S., Ma J., He D. (2017). Lightweight three-factor authentication and key agreement protocol for internet-integrated wireless sensor networks. IEEE Access.

[6] Jiang Q., Ma J., Yang C., Ma X., Shen J., Chaudhry S.A. (2017). Efficient end-to-end authentication protocol for wearable health monitoring systems. Comput. Electr. Eng..

[7] Raza M., Iqbal M., Sharif M., Haider W. (2012). A survey of password attacks and comparative analysis on methods for secure authentication. World Appl. Sci. J..

[8] Sin S.W., Zhou R., Li D., Isshiki T., Kunieda H. (2012). Narrow fingerprint sensor verification with template updating technique. IEICE Trans. Fundam..

[9] Lin C., Kumar A. (2018). Matching Contactless and Contact-Based Conventional Fingerprint Images for Biometrics Identification. IEEE Trans. Image Process..

[10] Parkhi O.M., Vedaldi A., Zisserman A. Deep Face Recognition. Proceedings of the British Machine Vision Conference.

[11] Ning X., Li W., Tang B., He H. (2018). BULDP: Biomimetic Uncorrelated Locality Discriminant Projection for Feature Extraction in Face Recognition. IEEE Trans. Image Process..

[12] Delac K., Grgic M. A survey of biometric recognition methods. Proceedings of the Elmar 2004. 46th International Symposium Electronics in Marine.

[13] Jacob R.J., Karn K.S. (2003). Eye tracking in human–computer interaction and usability research: Ready to deliver the promises. Mind Eye.

[14] Majaranta P., Bulling A. (2014). Eye tracking and eye-based human–computer interaction. Advances in Physiological Computing.

[15] Morimoto C.H., Mimica M.R. (2005). Eye gaze tracking techniques for interactive applications. Comput. Vis. Image Underst..

[16] Lv Z., Zhang C., Zhou B., Gao X., Wu X. (2018). Design and implementation of an eye gesture perception system based on electrooculography. Expert Syst. Appl..

[17] Karthikeyan S., Jagadeesh V., Shenoy R., Ecksteinz M., Manjunath B.S. From where and how to what we see. Proceedings of the 2013 IEEE International Conference on Computer Vision.

[18] Borji A., Itti L. (2013). State-of-the-art in visual attention modeling. IEEE Trans. Pattern Anal. Mach. Intell..

[19] Hansen D., Ji Q. (2010). In the eye of the beholder: A survey of models for eyes and gaze. IEEE Trans. Pattern Anal. Mach. Intell..

[20] Krafka K., Khosla A., Kellnhofer P., Kannan H., Bhandarkar S., Matusik W., Torralba A. (2016). Eye tracking for everyone. arXiv.

[21] Lotter W., Kreiman G., Cox D. (2016). Deep predictive coding networks for video prediction and unsupervised learning. arXiv.

[22] Komogortsev O.V., Karpov A., Holland C.D. (2015). Attack of mechanical replicas: Liveness detection with eye movements. IEEE Trans. Inf. Forensics Secur..

[23] Ali A., Deravi F., Hoque S. Spoofing attempt detection using gaze colocation. Proceedings of the 2013 International Conference of the BIOSIG Special Interest Group (BIOSIG).

[24] Zhang Y., Chi Z., Feng D. An Analysis of Eye Movement Based Authentication Systems. Proceedings of the International Conference on Mechanical Engineering and Technology.

[25] Saeed U. (2016). Eye movements during scene understanding for biometric identification. Pattern Recognit. Lett..

[26] Zhang Y., Bulling A., Gellersen H. Towards pervasive eye tracking using low-level image features. Proceedings of the Symposium on Eye Tracking Research and Applications.

[27] Zhang Y., Bulling A., Gellersen H. SideWays: A gaze interface for spontaneous interaction with situated displays. Proceedings of the SIGCHI Conference on Human Factors in Computing Systems.

[28] Zhang Y., Bulling A., Gellersen H. Pupil-canthi-ratio: A calibration-free method for tracking horizontal gaze direction. Proceedings of the 2014 International Working Conference on Advanced Visual Interfaces.

[29] Kumar M., Garfinkel T., Boneh D., Winograd T. Reducing shoulder-surfing by using gaze-based password entry. Proceedings of the 3rd symposium on Usable privacy and security.

[30] Weaver J., Mock K., Hoanca B. Gaze-based password authentication through automatic clustering of gaze points. Proceedings of the 2011 IEEE International Conference on Systems, Man, and Cybernetics.

[31] Bulling A., Alt F., Schmidt A. Increasing the security of gaze-based cued-recall graphical passwords using saliency masks. Proceedings of the SIGCHI Conference on Human Factors in Computing Systems.

[32] Boehm A., Chen D., Frank M., Huang L., Kuo C., Lolic T., Martinovic I., Song D. Safe: Secure authentication with face and eyes. Proceedings of the 2013 International Conference on Privacy and Security in Mobile Systems.

[33] De Luca A., Denzel M., Hussmann H. Look into my eyes!: Can you guess my password?. Proceedings of the 5th Symposium on Usable Privacy and Security.

[34] Kocejko T., Wtorek J. (2012). Gaze pattern lock for elders and disabled. Information Technologies in Biomedicine.

[35] Chen Y., Li T., Zhang R., Zhang Y., Hedgpeth T. EyeTell: Video-Assisted Touchscreen Keystroke Inference from Eye Movements. Proceedings of the EyeTell: Video-Assisted Touchscreen Keystroke Inference from Eye Movements.

[36] Sluganovic I., Roeschlin M., Rasmussen K.B., Martinovic I. Using reflexive eye movements for fast challenge-response authentication. Proceedings of the 2016 ACM SIGSAC Conference on Computer and Communications Security.

[37] Simonyan K., Zisserman A. Two-stream convolutional networks for action recognition in videos. Proceedings of the 27th International Conference on Neural Information Processing Systems.

[38] Wang L., Xiong Y., Wang Z., Qiao Y., Lin D., Tang X., Van Gool L. (2016). Temporal segment networks: Towards good practices for deep action recognition. Proceedings of the European Conference on Computer Vision.

[39] Ma C.Y., Chen M.H., Kira Z., AlRegib G. (2017). TS-LSTM and Temporal-Inception: Exploiting Spatiotemporal Dynamics for Activity Recognition. arXiv.

[40] Tesfaldet M., Brubaker M.A., Derpanis K.G. (2017). Two-stream convolutional networks for dynamic texture synthesis. arXiv.

[41] Abrams R.A., Meyer D.E., Kornblum S. (1989). Speed and accuracy of saccadic eye movements: Characteristics of impulse variability in the oculomotor system. J. Exp. Psychol. Hum. Percept. Perform..

[42] Zeiler M.D., Krishnan D., Taylor G.W., Fergus R. Deconvolutional networks. Proceedings of the 2010 IEEE Computer Society Conference on Computer Vision and Pattern Recognition.

[43] Zeiler M.D., Taylor G.W., Fergus R. Adaptive deconvolutional networks for mid and high level feature learning. Proceedings of the 2011 IEEE International Conference on Computer Vision.

[44] Zeiler M.D., Fergus R. Visualizing and understanding convolutional networks. Proceedings of the European Conference on Computer Vision.

[45] Long J., Shelhamer E., Darrell T. Fully convolutional networks for semantic segmentation. Proceedings of the IEEE Conference on Computer Vision and Pattern Recognition.

[46] Radford A., Metz L., Chintala S. (2015). Unsupervised representation learning with deep convolutional generative adversarial networks. arXiv.

[47] Cortes C., Vapnik V. (1995). Support-vector networks. Mach. Learn..

[48] Pedregosa F., Varoquaux G., Gramfort A., Michel V., Thirion B., Grisel O., Blondel M., Prettenhofer P., Weiss R., Dubourg V. (2011). Scikit-learn: Machine Learning in Python. J. Mach. Learn. Res..

[49] Lars B., Gilles L., Mathieu B., Fabian P., Andreas M., Olivier G., Vlad N., Peter P., Alexandre G., Jaques G. (2013). API design for machine learning software: Experiences from the scikit-learn project. ECML PKDD Workshop Lang. Data Min. Mach. Learn..

